# Tumour-Induced Osteomalacia—A Long Way to the Diagnosis Facilitated by [^68^Ga]Ga-DOTATATE PET/CT

**DOI:** 10.3390/jcm13061817

**Published:** 2024-03-21

**Authors:** Jolanta Kunikowska, Natalia Andryszak, Elżbieta Skowrońska-Jóźwiak, Kacper Pełka, Arkadiusz Zygmunt, Andrzej Lewiński, Marek Ruchała, Rafał Czepczyński

**Affiliations:** 1 Nuclear Medicine Department, Medical University of Warsaw, 02-091 Warsaw, Poland; 2 Department of Endocrinology, Metabolism and Internal Diseases, Poznan University of Medical Sciences, 61-701 Poznań, Poland; 3 Department of Endocrinology and Metabolic Diseases, Medical University of Lodz, 90-419 Łódź, Poland; 4 Department of Endocrinology and Metabolic Diseases, Polish Mother’s Memorial Hospital–Research Institute, 93-338 Łódź, Poland; 5 Laboratory of Centre for Preclinical Research, Department of Research Methodology, Medical University of Warsaw, 01-445 Warsaw, Poland

**Keywords:** tumour-induced osteomalacia (TIO), Ga-DOTATATE, osteogenic osteomalacia, phosphatonins, FGF23

## Abstract

**Background:** Tumour-induced osteomalacia (TIO) is a rare paraneoplastic syndrome. Detecting the primary tumour in TIO is challenging using conventional imaging methods. This study assesses the efficacy of [^68^Ga]Ga-DOTATATE PET/CT in identifying the primary tumour. **Methods:** Six patients with suspected TIO underwent [^68^Ga]Ga-DOTATATE PET/CT. The patients’ clinical history and biochemical parameters were analysed. **Results:** [^68^Ga]Ga-DOTATATE PET/CT successfully identified primary tumours in four patients (femoral bones for two, iliac bone for one, subcutaneous tissue of pubic region for one). Tumour removal led to clinical and laboratory improvement. In one patient, PET/CT showed rib uptake, but the biopsy was negative. One patient showed no tumour lesions on PET/CT despite clinical evidence. Two patients had focal recurrence at the primary tumour site, detected by follow-up PET/CT. **Conclusions:** [^68^Ga]Ga-DOTATATE PET/CT is a valuable tool for detecting primary tumours in TIO, aiding in accurate diagnosis and guiding surgery, leading to improved outcomes. Further research is needed to validate these findings and explore [^68^Ga]Ga-DOTATATE PET/CT in other paraneoplastic syndromes.

## 1. Introduction

Tumour-induced osteomalacia (TIO), also known as oncogenic osteomalacia, is a rare paraneoplastic syndrome reported in approximately 1000 cases worldwide [1]. However, due to unspecific symptoms and the lack of a global population-based epidemiology study, the number of cases is probably underestimated. 

TIO is caused by phosphaturic mesenchymal tumours (PMT), which secrete fibroblast growth factor 23 (FGF23) [1,2]. FGF23 is an endocrine hormone physiologically synthesized by osteocytes and osteoblasts and is involved in the regulation of serum phosphate concentration [3]. Similar to parathyroid hormone (PTH), FGF23 downregulates the reabsorption of phosphate from the proximal convoluted tubules in the kidneys [4]. Additionally, FGF23 inhibits PTH and 1.25-dihydroxyvitamin D (1.25(OH)_2_D) synthesis. The hypersecretion of FGF23 causes hyperphosphaturia, hypophosphataemia, and low or inappropriately normal 1.25(OH)_2_D levels, while calcium and PTH levels remain within the normal range [3]. 

Tumour-induced osteomalacia (TIO) clinically manifests with myalgia, bone pain, osteomalacia, and fragility fractures [5]. These symptoms are nonspecific, leading to misdiagnosis and incorrect treatment in over 95% of cases [1]. The median time to diagnosis is 2.9 years, but delays in diagnosis as long as 26 years have been reported [5,6]. Diagnosis relies heavily on a combination of laboratory and imaging assessments.

Patients with TIO typically exhibit hypophosphataemia, hyperphosphaturia, and elevated FGF23 levels, prompting clinicians to incorporate bone imaging modalities to identify the mesenchymal tumour responsible for these laboratory abnormalities. Complete body scanning, encompassing the entire head and distal extremities where tumours are often found, is essential for tumour localization. Traditional imaging methods struggle to accurately pinpoint tumour locations.

Precise tumour localization is crucial, given that surgical resection is deemed the only effective treatment for TIO. Imaging for TIO necessitates a blend of functional techniques that detect cellular metabolic activity and subsequent anatomical imaging to precisely define the tumour’s location.

Functional imaging techniques involve single-photon emission computed tomography or positron emission tomography with computed tomography (SPECT/CT and PET/CT) in combination with various radioactive compounds, to identify lesions with high metabolic activity. ^18^F-fluorodeoxyglucose ([^18^F]FDG) PET/CT is widely used in the management of patients with malignancies. However, [^18^F]FDG is not specific to neoplastic lesions and may indicate other areas of high metabolic activity, like inflammation, infection, or active fracture healing [7]. That is why it is important to indicate radionuclides that are specific only to TIO cells. A recent meta-analysis has shown that somatostatin-receptor-based imaging with [^68^Ga]Ga-DOTATATE PET/CT has higher sensitivity than [^18^F]FDG PET/CT in the detection of TIO [8]. El-Maouche et al. [9]. prospectively compared [^68^Ga]Ga-DOTATATE PET/CT to [^18^F]FDG PET/CT and proved significantly greater sensitivity and specificity, suggesting that [^68^Ga]Ga-DOTATATE PET/CT may be the best imaging modality for TIO localization [10].

The aim of this paper is to present the role of [^68^Ga]Ga-DOTATATE PET/CT as an imaging tool used to detect primary tumours in a real-life clinical scenario as well as to evaluate the treatment outcomes in patients with TIO. Below, we present six cases of the clinical application of [^68^Ga]Ga-DOTATATE PET/CT in patients with a strong suspicion of TIO.

## 2. Materials and Methods

All procedures were in accordance with the ethical standards of the institutional and/or national research committee and with the Declaration of Helsinki and its later amendments or comparable ethical standards. This single-institution study was approved by the Ethical Committee of the Medical University of Warsaw (KB/224/2010). Written informed consent was obtained from all patients according to institutional guidelines.

### 2.1. Patients

A total of 6 consecutive patients (3 females, 3 males, median age 48.8 years) with a clinical and biochemical suspicion of TIO were studied. A total of 14 PET/CT examinations were performed in 6 patients who were referred to the nuclear medicine department of a tertiary referral hospital.

### 2.2. Data Collection

Detailed information on demographics and clinical history, including biochemical parameters, tumour site, and detection methods were recorded.

### 2.3. Biochemical Parameters

The panel of biochemical tests including alkaline phosphatase (ALP), calcium (Ca), 25OHD, 1.25(OH)_2_D, estimated glomerular filtration rate (eGFR), fasting serum phosphate, PTH, fractional excretion of phosphate, FGF23, prior to tumour resection and during follow-up was collected. Values of the tubular reabsorption of phosphate (TRP) <0.82 were considered as indicative of hyperphosphaturia [11].

### 2.4. [^68^Ga]Ga-DOTATATE PET/CT Imaging

The labelling procedure adhered to previously established protocols (10). A comprehensive whole-body PET/CT scan, spanning from the vertex to the upper thigh, was conducted utilizing a Biograph 64 TruePoint PET/CT scanner manufactured by Siemens Medical Solutions in Knoxville, US. Imaging data were acquired 60 min post-injection of [^68^Ga]Ga-DOTATATE, with an administered activity of 2 MBq/kg of body weight.

To enhance renal washout, 20 mg of furosemide was promptly administered following the injection of [^68^Ga]Ga-DOTATATE, and patients were directed to consume 1.5 L of water. Furthermore, patients were instructed to void the bladder prior to the commencement of the PET/CT examination.

The patients were placed in a supine position with their arms raised, according to standard CT practice. A continuous CT was acquired in the spiral mode using 120 kV, 170 mAs, 2 mm slice thickness, and a pitch of 0.8. The patient position and the area of PET/CT examination were identical to that of CT, at a rate of 3 min per bed position, 6–7 bed positions, depending on the patient’s height.

The emission data of both examinations was reconstructed on a 168 × 168 matrix, using an ordered subset expectation maximisation algorithm (3 iterations, 21 subsets). The attenuation was corrected with CT data. The PET/CT images, which consisted of whole-body attenuated and non-attenuated PET, CT, and fused images, were transferred to a multimodal workstation (MMWS) (Syngo TrueD Siemens Medical Solutions) for analysis.

### 2.5. Image Analysis

Image analysis was performed using the Siemens MMWS. PET/CT fusion images were analysed using a vendor-provided analysis software package (TRUE D, Siemens Medical Solutions, 1.0 software). In the visual analysis, the [^68^Ga]Ga-DOTATATE PET/CT findings were designated as positive when focal uptake was seen in the coronal, transaxial, and sagittal views. Linear or tubular areas of increased uptake in the intestinal tract were described as physiologic and negative for malignancy.

For the quantitative analysis, the maximal standard uptake value (SUVmax) of each positive lesion was measured on both PET/CT images with spherical volumes of interest (VOIs) with a diameter adapted to the lesion’s size. Attenuation-corrected PET images and PET/CT images were analysed. The PET images without attenuation correction were also reviewed.

## 3. Results

### 3.1. Patient Characteristics

This retrospective case series includes six patients (three males and three females aged 48.8 ± 7.2), diagnosed with TIO between January 2011 and April 2023. During this time, five out of six patients had more than one [^68^Ga]Ga-DOTATATE PET/CT examination.

Clinically, all patients reported bone pain, muscle weakness/fatigue, and mobility difficulties. In five out of six cases, multiple fragility fractures were observed. All patients received several misdiagnoses (osteoporosis, rheumatic, or neurological disease) and the final diagnosis of TIO was made with a median delay of 56.4 months (range: 42–70 months). In one patient diagnosed with TIO, the tumour has not been found. Serum biochemistry results revealed severe hypophosphataemia, significantly increased alkaline phosphatase levels, and normal calcium levels. PTH was slightly elevated in two out of six patients. In four out of six patients, 1.25(OH)_2_D was decreased. The concentration of FGF23 was increased in all reported cases except one patient whose FGF23 measurement was not available. Detailed biochemical parameters are shown in Table 1.

### 3.2. Other Imaging

Prior to [^68^Ga]Ga-DOTATATE PET/CT examination, no other imaging studies revealed tumour location. All patients underwent bone scintigraphy with the use of [^99m^Tc]Tc-MDP, which showed diffuse increased uptake in the bone with a typical pattern of osteomalacia. Radiography and CT showed multiple bone fractures in five out of six cases. Densitometry revealed reduced bone mineral density (BMD) within the range of osteoporosis in all patients.

### 3.3. Somatostatin Receptor Imaging

[^68^Ga]Ga-DOTATATE PET/CT indicated focal uptake in the majority of patients in bones: two patients in the femur with SUVmax values of 7.2 and 14.2 and one in the iliac bone with an SUVmax of 4.3. One patient had focal uptake in the soft tissue in the pubic area with an SUVmax of 40.8.

One examination revealed suspicious uptake in the second right rib (SUVmax 3.6); however, the biopsy was negative for malignancy. The patient underwent [^18^F]FDG PET/CT, which did not reveal any suspicious lesions. Likewise, the follow-up [^68^Ga]Ga-DOTATATE PET/CT examination, performed 6 months later, was negative.

Two patients had a relapse of the disease after surgery. [^68^Ga]Ga-DOTATATE PET/CT examination revealed focal recurrence with SUVmax values of 2.6 and 14.3, in the same location as the primary tumour.

In one patient, despite clinical evidence of TIO, two [^68^Ga]Ga-DOTATATE PET/CT scans did not reveal any suspicious lesions that could indicate a mesenchymal tumour.

### 3.4. Case Presentation

#### 3.4.1. Case 1

A 44-year-old man, with a 4-year history of generalised bone pain, multiple fragility fractures (fractures of the spinous processes of the Th1, 2, 5, 6, 12, right and left ribs, and left scapula) and muscle weakness, was referred for [^68^Ga]Ga-DOTATATE PET/CT examination. In the entire symptomatic period, the patient had been consulted by an orthopaedic surgeon, neurologist, and rheumatologist. The rheumatologic blood tests as well as the borreliosis test were negative. Over the years, the patient underwent several imaging examinations including bone scintigraphy and magnetic resonance (MR). The bone scintigraphy showed diffuse increased uptake with the suggestion of osteomalacia. Initial tests showed slightly elevated levels of PTH—75.7 pg/mL (normal range: 15–65 pg/mL), calcium—2.5 mmol/L (2.1–2.6 mmol/L), and eGFR—100 mL/min/1.73 m^2^, an increased level of alkaline phosphatase (ALP)—237 IU/L (30–120 IU/L), and reduced levels of phosphate—0.55 mmol/L (0.8–1.5 mmol/L), and 25(OH)D—17.6 ng/mL.

Further investigations showed persistent hypophosphataemia with high renal phosphate wasting (phosphate excretion 87 mmol/24 h, normal: 12–42 mmol/24 h), high FGF23—423 RU/mL (0–100 RU/mL), and signs of severe osteoporosis in densitometry.

**Figure 1:**
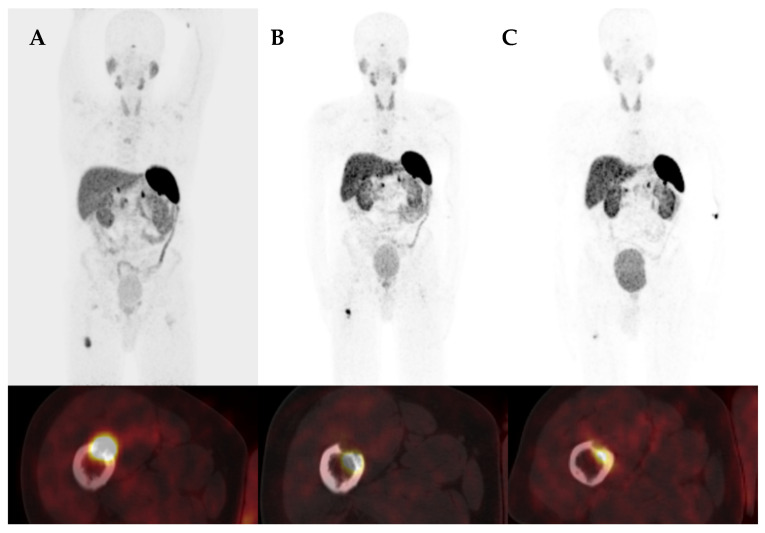
[^68^Ga]Ga-DOTATATE PET/CT images of a 44-year-old man with a near 4-year history of osteoporosis and hypophosphataemia with multiple bone fractures (Case 1). The [^68^Ga]Ga-DOTATATE PET/CT images showed intense focal uptake in the right femur with an SUVmax of 14.2 (**A**). The follow-up [^68^Ga]Ga-DOTATATE PET/CT revealed focal recurrence with an SUVmax of 14.3 (**B**). Thermoablation was the next step, but phosphate was still below the normal level. The subsequent [^68^Ga]Ga-DOTATATE PET/CT revealed a focal recurrence with an SUVmax of 7.9 (**C**). Upper row—MIP images, bottom row—fused transaxial PET/CT images of the right femur.

During the 4-year period before diagnosis, the patient was intermittently taking different doses of vitamin D, calcitriol, and phosphate with no improvement in densitometry. Finally, the TIO suspicion was raised by an endocrinologist. The patient was referred for [^68^Ga]Ga-DOTATATE PET/CT examination, which revealed multiple foci of increased uptake in the rib fractures, as well as high focal uptake in the left femur suggestive of the primary tumour location (SUVmax 14.2). MR of the femur showed a tumour in the same location without bone marrow infiltration. The lesion was surgically removed, and the histopathology confirmed a mesenchymal tumour. Unfortunately, after surgery, persistent hypophosphataemia was present with only a little improvement from 0.55 to 0.70 mmol/L. A follow-up [^68^Ga]Ga-DOTATATE PET/CT scan showed incomplete tumour resection. The patient underwent thermoablation, but the phosphate level was constantly decreased (0.76 mmol/L). Subsequent [^68^Ga]Ga-DOTATATE PET/CT examination showed increased uptake in the remaining tumour (Figure 1). Finally, the orthopaedic surgeon decided to remove part of the bone, including the tumour, with simultaneous reconstruction using screws. After the second surgery, phosphorus supplementation was discontinued. During follow-up, 1-year post-surgery, the patient was asymptomatic, and all biochemical tests remained within the normal range, including phosphate—1.0 mmol/L and FGF23—80 RU/mL. Due to the pronounced and long-lasting osteoporosis and spinal fractures due to hypophosphataemia, a 9 cm height reduction was observed.

#### 3.4.2. Case 2

A 55-year-old patient with a near 7-year history of generalized weakness, especially within the lower limbs (inability to move unaided, the need to use a wheelchair, and the help of a caregiver even with simple activities such as dressing or washing) and numerous low-energy fractures of the Th6–L5 vertebrae (the patient’s height decreased by 25 cm) and ribs, was diagnosed with dramatic hypophosphataemia (0.3 mmol/L n: 0.87–1.45). At the time of diagnosis, the patient did not present with increased hyperphosphaturia (13.2 mmol/24 h—n: 12.9–42.0), while tubular reabsorption indicated renal loss of phosphorus (TmP/GFR—0.57; TRP = 55%), which confirms that 24 h phosphate excretion is the resultant of the phosphorus load in the primary urine filtrate (if the serum phosphorus concentration is very low, hyperphosphaturia may be absent), making the analysis of tubular phosphate resorption much more sensitive. The bone scintigraphy in 2008 indicated features of metabolic remodelling typical for osteomalacia. The patient received long-term phosphate and active and native vitamin D supplementation, which caused a significant improvement in the general condition of the patient (he was able to move independently) and significant increase in BMD. However, persistent hypophosphataemia (0.55 mmol/L), elevated alkaline phosphatase (447 U/L), and hyperphosphaturia (56.2 mmol/24 h) were still present. No classical imaging studies (CT, MRI) were able to visualize the location of the tumour. The [^18^F]FDG PET/CT was performed and revealed increased uptake in the mediastinal lymph nodes (SUVmax 6.2) and pubic bone (SUVmax 3.0) as well as multiple fragility fractures of the ribs. Nevertheless, [^18^F]FDG PET/CT did not indicate the primary tumour causing hypophosphataemia. Finally, in 2011, the patient underwent a [^68^Ga]Ga-DOTATATE PET/CT examination, which revealed uptake in the head of the left femur with the SUVmax of 7.2. The patient underwent surgical resection of the tumour with clinical improvement. He did not require further pharmacological therapy. 

#### 3.4.3. Case 3

**Figure 2:**
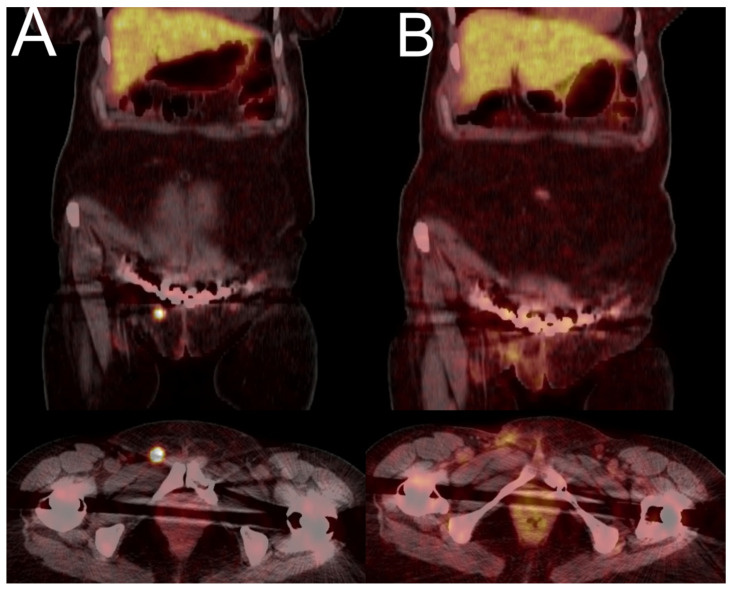
[^68^Ga]Ga-DOTATATE PET/CT images of a 58-year-old woman with a near 4-year history of osteoporosis and hypophosphataemia (Case 3). The [^68^Ga]Ga-DOTATATE PET/CT images show intense focal uptake in the soft tissue of the pubic region with an SUVmax of 40.8 (**A**). After surgery, phosphatase slightly increased. The follow-up [^68^Ga]Ga-DOTATATE revealed a focal recurrence with an SUVmax of 2.6 (**B**). Upper row—fused frontal PET/CT images, lower row—fused transaxial PET/CT images of the pelvis.

A 58-year-old woman presented with symptoms of bone pain and mobility difficulties for 60 months prior to diagnosis. The patient experienced multiple bone fractures (two fractures of the pelvis, fractures of both hips, ribs, and left foot), and underwent a pelvic fracture operation and bilateral femoral neck endoprosthesis. Due to densitometric signs of osteoporosis, she was treated with alendronate and vitamin D supplementation for almost 3 years, without any improvement. In 2011, the patient was referred to the hospital due to severe bone pain, not responsive to non-steroidal anti-inflammatory drugs and tramadol, and deterioration of walking capability. Initially, the PTH concentration was slightly elevated and 25OHD and 1.25(OH)_2_D levels were decreased. After discovering significant hypophosphataemia, increased tubular reabsorption of phosphate, as well as increased alkaline phosphatase, the suspicion of TIO was raised, confirmed later by elevated FGF23 levels (406 RU/mL). Therapy with oral phosphates and active vitamin D led to walking improvement and reduction of pain. The CT and MR scans of the chest and abdomen did not indicate any pathological lesions, but the [^68^Ga]Ga-DOTATATE PET/CT (Figure 2) revealed increased uptake in the adipose tissue of the mons pubis (SUVmax 40.8). Despite resection of the tumour, she still required phosphate supplementation. After 6 months, the follow-up [^68^Ga]Ga-DOTATATE PET/CT was performed and indicated local recurrence/remnant of the tumour with the SUVmax of 2.6. The patient was reoperated but she still required phosphate and active vitamin D supplementation. After the second surgery, severe complications of wound healing occurred, but after healing she did not require either phosphates or vitamin D supplementation. Two years after the second surgery, a follow-up [^68^Ga]Ga-DOTATATE PET/CT was negative for pathological uptake and the patient was free from symptoms. 

#### 3.4.4. Case 4 

A 42-year-old woman complained of severe pain in the lower back and lower extremities resistant to non-steroidal analgesics for 5 years. Due to mobility difficulties, she moved on crutches. Since malignancy was suspected, various imaging examinations were performed. MR, RTG, and CT indicated discopathy and multiple healed fractures. Scintigraphy revealed remodelling typical for osteomalacia. Low BMD was found in densitometry. In 2011, hypophosphataemia and hypophosphaturia, accompanied by high concentration of ALP were found. Therapy with oral phosphates and active vitamin D was initiated, with marked clinical improvement, reduction of pain, and improvement of mobility; however, despite supplementation, the hypophosphataemia persisted. Due to the suspicion of TIO, the patient underwent numerous imaging studies, but they did not reveal the location of the tumour. Additionally, the first [^68^Ga]Ga-DOTATATE PET/CT examination indicated no pathological uptake. After two years of follow-up, an increase in FGF23 to 686 RU/mL was found and [^68^Ga]Ga-DOTATATE PET/CT re-examination showed tracer uptake in the posterior part of the left hip acetabulum (SUVmax 7.8). Unfortunately, the patient denied surgery and moved to another country.

#### 3.4.5. Case 5 

A 45-year-old woman presented with unspecific bone pain and mobility difficulties. Serum biochemistry revealed hypophosphataemia (0.65 mmol/L), increased ALP (165 U/L) and PTH, as well as vitamin D within the normal range. The scintigraphy examination showed features of metabolic remodelling typical for osteomalacia. The [^68^Ga]Ga-DOTATATE PET/CT indicated suspicious uptake in the 2nd right rib (SUVmax 3.6); however, the biopsy was negative for malignancy. The patient underwent [^18^F]FDG PET/CT, which did not reveal any suspicious lesions. Another [^68^Ga]Ga-DOTATATE PET/CT examination was performed two years later and it showed no radiotracer uptake either. 

#### 3.4.6. Case 6

**Figure 3:**
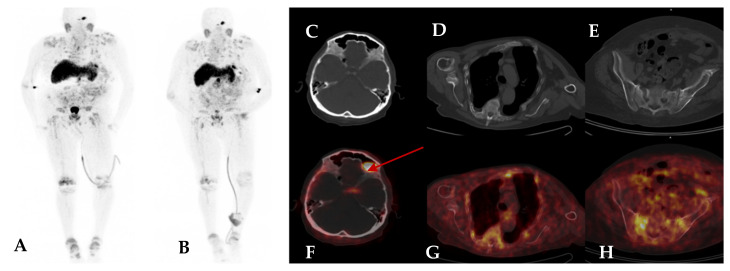
[^68^Ga]Ga-DOTATATE PET/CT images of a 66-year-old man with a 3.5-year history of osteoporosis and hypophosphataemia with multiple bone fractures (Case 6). The [^68^Ga]Ga-DOTATATE PET/CT (**A**) showed intense focal uptake in a meningioma in the left frontal sinus (SUVmax 21.1, red arrow), previously detected by MR, as well as in multiple bone fractures (with SUVmax up to 9.8), but no primary tumour was found ((**A**)—MIP image; (**C**–**E**)—CT images; (**F**–**H**)—transaxial PET/CT images). One year later, the follow-up [^68^Ga]Ga-DOTATATE PET/CT was nearly the same, except for some bone reparation process ((**B**)—MIP image).

A 66-year-old man presented with a 3-year clinical history consistent with hypophosphataemic osteomalacia, accompanied by multiple spontaneous bone fractures, along with confirmed impairment of renal phosphate reabsorption. Despite various imaging tests, including scintigraphy, [^18^F]FDG PET/CT, and [^68^Ga]Ga-DOTATATE PET/CT, no obvious features of a mesenchymal tumour were observed. The patient had numerous pathological, idiopathic fractures in the area of the pelvis, ribs, and spine. In addition, the patient had severe problems with walking, a loss of height by 13 cm was observed, and he underwent bilateral hip joint and right knee joint replacement. The ALP was elevated up to 588 U/L (normal range: 40–129), while the patient presented with hypophosphataemia (0.45 mmol/L, normal: 0.81–1.45) and hyperphosphaturia. The levels of calcium, PTH, calcitonin, and vitamin D were within the normal range. CT scans of the neck, spine, and pelvis indicated multiple pathological fractures. The patient underwent [^18^F]FDG PET/CT which did not reveal any suspicious uptake. The patient underwent two [^68^Ga]Ga-DOTATATE PET/CT scans which did not reveal a definitive focal point indicating the location of a mesenchymal tumour (Figure 3). In both examinations, numerous foci of increased tracer uptake were visible in the bones (some with higher, some with the same, and some with lower tracer accumulation), mainly in the spine, shoulder girdle bones, pelvic bones, calcaneus, and metatarsal bones. However, the increased focal uptake corresponded to fracture clefts on the CT scan, that was most likely associated with the healing process. Furthermore, the [^68^Ga]Ga-DOTATATE PET/CT scans indicated focal tracer accumulation in the left frontal region, which corresponded to the meningioma previously reported in the MR examination.

The patient continued to receive phosphate supplementation and alfacalcidol as treatment with improvement; however, the mesenchymal tumour was never found.

## 4. Discussion

A series of six patients diagnosed with TIO originating from one diagnostic centre has been presented here. Although the number of patients is small, it is worth mentioning that approximately 1000 cases have been reported in the medical literature so far [1], including only five single reports from Poland [12,13,14,15,16,17]. All patients presented in this study demonstrated similar clinical signs including severe pain, proximal muscle weakness, fractures, loss of height, and deterioration in mobility. As the clinical presentation is not characteristic and the disease is uncommon, a significant delay in diagnosis occurred. Due to low BMD and the occurrence of fractures, patients were most often misdiagnosed with osteoporosis. According to the literature, 80% of patients experienced a diagnostic delay longer than 2 years [18]. In this case series, an average delay in diagnosis was as much as 56.4 months, which is longer than the 43 months previously reported in the literature [19]. Typically for TIO, all patients demonstrated low serum phosphate levels [1,2,18]. Unfortunately, serum phosphate level is not a routine blood test in patients suspected to have osteoporosis or metabolic bone disease, which was one of reasons for the delayed diagnosis. 

Hypophosphataemia in TIO is a result of the phosphaturic effect of FGF23; however, most patients from the presented series demonstrated normal loss of phosphate in 24 h urine collection in contrast to significantly lowered tubular reabsorption of phosphates (TRP), that has been described as a more sensitive parameter of renal phosphate wasting than phosphaturia [11]. 

All patients demonstrated significantly elevated levels of ALP, typical for osteomalacia of different origins. Surprisingly, two patients showed slightly elevated PTH levels with normal calcium, while the typical biochemical pattern for TIO includes both normal serum calcium and PTH. Increased PTH concentration might reflect secondary hyperparathyroidism caused by low levels of vitamin D and calcitriol [1]. It should be emphasized that abnormalities of PTH concentrations did not correspond to the severity of clinical symptoms. 

After establishing the diagnosis of TIO, the main challenge was to localize the FGF23-producing tumour. External tumours can be easily detected by palpation [13], but in the case of an internal location, imaging is necessary. Conventional imaging is frequently ineffective, because the tumours can be small and can develop anywhere in the body [18]. This case series demonstrated the usefulness of [^68^Ga]Ga-DOTATATE PET/CT in localizing internal tumours causing TIO in contrast to negative findings, obtained in many other imaging techniques (scintigraphy, MR, CT, USG). The effectiveness of [^68^Ga]Ga-DOTATATE PET/CT in TIO diagnostics was reported previously. Filipova et. al. presented a case of a 53-year-old woman with a 10-year history of symptoms [20]. Using [^68^Ga]Ga-DOTATATE PET/CT, the TIO tumour was found in the L2 vertebral body [20]. The CT and MR results visualised an osteolytic tumour, but only the PET/CT scan could characterize the pathology of the lesion. Another larger study presented twenty-two lesions of TIO in nine patients and confirmed the advantage of [^68^Ga]Ga-DOTATATE over conventional imaging and [^18^F]FDG PET/CT in the detection of TIO lesions [21].

Breer et al. presented the cases of five patients with TIO, who underwent both ^111^Indium-octreotide scintigraphy SPECT/CT as well as ^68^Ga DOTATATE PET/CT for tumour detection [22]. The SPECT/CT indicated the TIO only in one out of five patients, whereas PET/CT revealed the primary tumour location in all cases and the surgical resection could be performed. 

Recently, Hou et al. presented a comparison of the diagnostic accuracy of a new radiopharmaceutical ^68^Ga-DOTA-JR11, a somatostatin-receptor-subtype-2-specific antagonist, and the routinely employed [^68^Ga]-DOTATATE PET/CT in TIO imaging. The ^68^Ga-DOTATATE presented a higher sensitivity, as it indicated the tumour in 18 of 19 cases of TIO, while the ^68^Ga-DOTA-JR11 was positive only in 11 of 19 patients [10].

In addition, we have observed the case of increased false-positive uptake in [^68^Ga]Ga-DOTATATE PET/CT examination mimicking the TIO lesion. This corresponds to a previously reported case, which indicated increased uptake of the tracer in inflammatory foci and bone fractures [23]. Therefore, somatostatin receptor (SSTR) expression may also be observed in inflammatory/granulomatous conditions and fractures/degenerative bone disease leading to false-positive scan findings.

As we presented in our paper, the [^68^Ga]Ga-DOTATATE PET/CT can be regarded as an imaging modality of choice in TIO, as it unambiguously revealed tumour location in four out of five patients. In the first presented case, only [^68^Ga]Ga-DOTATATE PET/CT showed bone marrow infiltration, which was not visible on MR. In each of the presented cases, no other imaging modality was able to identify the location of the primary tumour, which was crucial for a successful surgery. Moreover, the [^68^Ga]Ga-DOTATATE PET/CT could be used as a follow-up imaging modality, as in the two presented cases, it was able to indicate local recurrence after surgery. It should be underlined that [^68^Ga]Ga-DOTATATE PET/CT was not applied in the previously published cases from Poland; tumour localization was made by palpation [13], [^18^F]FDG-PET/CT [14], MR, and CT [15], as well as the somatostatin receptor scintigraphy [16]. It is noteworthy that for most of the described cases, the tumours were located in the pelvic region, while data from the literature reported tumours located in the pelvis in only 10.3% of patients [18]. 

The current study has major limitations to be disclosed. This is a retrospective series of cases referred to a single diagnostic centre. The number of patients is therefore limited. Subsequent to the casuistic occurrence of this condition, the time in which the patients were referred for imaging was extremely long. Nevertheless, the diagnostic methodology (i.e., radiopharmaceutical, PET/CT scanner, and acquisition parameters) did not change over the years. To overcome the limitations of the single-centre study design, the results should be verified in a multicentre setting and/or summarised in a meta-analysis.

## 5. Conclusions

Our data suggest that in the case of a high suspicion of TIO, the [^68^Ga]Ga-DOTATATE PET/CT examination should be considered as the most accurate and useful imaging method. TIO may present with recurrence after surgical treatment and the [^68^Ga]Ga-DOTATATE PET/CT is also able to adequately indicate a focus of recurrence. The incidence of TIO is underestimated, and delayed diagnosis causes serious health consequences, so further efforts to raise awareness of TIO are recommended. 

## Figures and Tables

**Table 1:** Characteristics of patients at diagnosis. TRP—tubular reabsorption of phosphate, PTH—parathormone, FGF—fibroblast growth factor, DXA—dual-energy X-ray absorptiometry.

	Patient 1	Patient 2	Patient 3	Patient 4	Patient 5	Patient 6
Age [years]	44	55	58	42	45	66
Gender	M	M	F	F	F	M
Delay of diagnosis [months]	42	70	60	65	45	3
Serum phosphate N: 0.87–1.45 mmol/L	0.47	0.30	0.51	0.57	0.65	0.45
Urine phoshate N: 12.9–40.2 mmol/L	56.4	13.26	21.96	18.72	66	>40
TRP N: 85–95%	Not assessed	57	58	63	70	Not assessed
PTH N: 15–65 pg/mL	75	28	92	50	45	57
Serum calcium N: 2.12–2.55 mmol/L	2.13	2.12	2.33	2.32	2.2	2.37
Alkaline phosphatase N: 38–126 U/L	165	447	245	294	237	588
FGF23 N: <100 RU/mL	423	248	406	686	Not assessed	318
Densitometry	DXA spine T-score—3.6 DXA hip T-score total—3.3, neck—3.3	DXA hip: Tsc neck—6.0 total—5.8 DXA of spine was not assessed due to fractures	DXA spine T-score—3.5 DXA hip was not assessed due to bilateral endoprosthesis	DXA spine T-score—3.7 DXA hip T-score— neck—4.2; total—4.3	Osteoporosis: details are not accessible	DXA spine T-score:—0.8 DXA T-score for 33% of the distal forearm:—0.2

## Data Availability

The data presented in this study are available on request from the corresponding author.
